# Intracameral voriconazole for severe fungal keratitis: a case series

**DOI:** 10.5935/0004-2749.2024-0207

**Published:** 2024-12-26

**Authors:** Fernanda M. Bezerra, Ludmila N. P. Silva, Larissa L. Aguiar, Maria Cecília Z. Yu, Flavio J. Rocha, Luciene B. Sousa, Ana Luisa Höfling-Lima, Lauro A. de Oliveira

**Affiliations:** 1 Department of Ophthalmology and Visual Sciences, Escola Paulista de Medicina, Universidade Federal de São Paulo, São Paulo, SP, Brazil; 2 Department of Ophthalmology, Faculdade de Medicina, Universidade Federal de Uberlândia, Uberlândia, MG, Brazil; 3 Fundação Banco de Olhos de Goiás, GO, Brazil

**Keywords:** Antifungal agents, Fungi, Corneal transplantation, Keratitis, Eye infections, fungal, Voriconazole

## Abstract

**Purpose:**

This study aimed to report the use, efficacy, and safety of intracameral voriconazole as an adjuvant treatment for deep fungal keratitis.

**Methods:**

This was a prospective case series of seven eyes with fungal keratitis with anterior chamber involvement or a corneal ulcer refractory to conventional topical treatment. In addition to topical treatment with 0.15% amphotericin B eye drops, voriconazole 50 µg/ 0.1 mL was administered to the anterior chamber of each affected eye up to four times within 72 h. The primary outcome measures were healing (fungal eradication) and the need for therapeutic keratoplasty. Best-corrected visual acuity was a secondary outcome measure.

**Results:**

Three cases were confirmed by confocal microscopy, and four were diagnosed from positive culture tests. At presentation, one patient had a best-corrected visual acuity of 20/80, while all others had hand motion or worse. Four cases received one intracameral injection, two cases received three, and one case received four injections. There were no complications after any of the intracameral voriconazole injections. Four patients had imminent corneal perforations and were treated with cyanoacrylate adhesive and bandage contact lenses. Four patients recovered from the infection, and three underwent therapeutic keratoplasty. The final best-corrected visual acuity was improved in two cases but all patients had a final visual acuity of counting fingers or worse.

**Conclusion:**

As an adjuvant treatment for deep fungal keratitis, intracameral voriconazole injection is a feasible option. Although fungal eradication was achieved in all patients, three required therapeutic keratoplasty and all patients had unsatisfactory visual acuity outcomes.

## INTRODUCTION

Infectious keratitis is a major cause of monocular blindness worldwide^(1)^. Ocular surface disorders, refractive surgery, ocular trauma, and the widespread use of topical steroids and broad-spectrum antibiotics have all contributed to an increased prevalence of fungal infection^(2^,^3)^.

Regardless of the causative agent, topical drugs are the preferred treatment option due to ease of administration, patient adherence, and good responses in the early stages of infection. However, the poor penetration of many topical drugs makes them unsuitable treatments for deep corneal infiltrates. This makes fungal keratitis, particularly cases with deeper fungus growth, challenging to treat. While fungal keratitis is less prevalent than bacterial keratitis, it accounts for approximately half of the cases of microbial keratitis that require therapeutic keratoplasty^(4^,^5)^. It is estimated that 12-38% of patients with fungal keratitis require transplantation. Despite the *in vitro* susceptibility and adequate clinical treatment, several factors can impede fungal eradication, including antifungal bioavailability and biofilm formation^(6)^.

The usual initial treatment for filamentous fungi is 5% natamycin, while yeasts are treated with 0.15% amphotericin B. Both of these drugs are polyene macrolides with limited corneal and ocular penetration capabilities. Therefore, research has begun to investigate alternative methods of administration such as intracameral and intrastromal. These approaches may be of use as adjuvants to topical applications. Such targeted drug delivery is a promising means of improving the concentration and bioavailability of antifungals^(7^-^9)^.

Newer antifungals such as voriconazole, posaconazole, and caspofungin, have shown improved efficacy, safety, and better corneal penetration than their older counterparts^(10)^. Voriconazole, a new-generation triazole, has gained popularity in ophthalmological appli-cations due to its broad spectrum, depth of ocular penetration, and low toxicity^(7^-^11)^.

Although topical voriconazole in patients with filamentous fungal keratitis, particularly *Fusarium* sp., is associated with worse outcomes than topical natamycin treatment^(12)^, the inefficacy of conventional treatments for deep fungal keratitis has generated interest in the targeted delivery of voriconazole. In this study, we present seven patients with deep or refractory fungal keratitis who were treated with adjuvant intracameral voriconazole.

## METHODS

This prospective study was conducted at the Department of Ophthalmology and Visual Sciences, Federal University of São Paulo (UNIFESP), Brazil. It was approved by the UNIFESP Ethics Committee (approval number 00515318.0.0000.5505) and conducted in accordance with the tenets of the 2013 revision of the Declaration of Helsinki. All participants provided written informed consent to participation.

Patients clinically diagnosed with fungal keratitis presenting with anterior chamber involvement or corneal ulcers who did not respond to conventional topical antifungal treatment were included in this study. Samples from all patients were taken and cultured. Corneal scrapings were obtained under topical anesthesia. These were smeared for potassium hydroxide (KOH) and Gram staining was performed. They were then inoculated in blood agar, chocolate agar, Sabouraud agar, and thioglycolate broth. The samples with culture-negative results underwent confocal microscopy. Patients who tested positive for bacteria or acanthamoeba were excluded from this study.

During the first examination, each patient underwent a detailed anamnesis, a visual acuity test, slit-lamp biomicroscopy, and, if possible, fundoscopy. Patients were examined every 48 h until they began to respond favorably to treatment. The size and depth of infiltrates and the heights of hypopyons were measured during follow-up visits. Slit lamp images were used to document the ophthalmic evaluations.

All patients received a topical 0.15% amphotericin B regimen for ongoing administration, initially hourly. An intracameral voriconazole injection (50 µg/0.1 mL) fractioned by Eye Pharma^®^ (São Paulo, SP, BR) ophthalmic pharmacy was performed when the patient was unresponsive to topical treatment. The procedure was performed in an operating room under topical or peribulbar anesthesia. Voriconazole (50 µg) was administered after paracentesis and slight decompression of the anterior chamber. If there was no or little improvement, up to four intracameral injections were administered 72 h apart. Patients with imminent corneal perforations were treated with cyanoacrylate glue and bandage contact lenses. Therapeutic keratoplasty was recommended for larger perforations and in those refractory to the voriconazole treatment. Cure was defined as complete infection resolution. Topical antifungal treatment was continued for at least a week, and all patients were followed up for a minimum of 3 months after treatment ended.

## RESULTS

Seven eyes of seven patients were included in this study, with a mean age of 53.1 (32-79) years. The sample comprised four males and three females. Ocular trauma (four cases) and previous keratoplasty with topical corticosteroid use (one case) were risk factors for the development of keratitis. There were four culture-proven cases and three cases with positive confocal microscopy results. The positive cultures revealed *Fusarium solani* in two patients, *Scopulariopsis brevicaulis* in one patient, and *Candida parapsilosis* in one patient. The confocal microscopy images showed fungal hyphae in two cases and pseudohyphae in one. Before treatment with intracameral voriconazole, all eyes had corneal ulcers with deep stromal infiltration and/or anterior chamber involvement.

Table 1 presents the clinical features and demographics of the patients. Full-thickness infiltrate was present in six patients, endothelial plaque in four patients, and hypopyon in four patients. All of the patients had previously received treatment for other etiologies and used medications for varying lengths of time, as shown in Table 1. Once fungal etiology was suspected, all patients were started on topical 0.15% amphotericin B, which was applied hourly for at least 1 week (Cases 2, 3, and 4 for one week and Cases 1, 5, 6, and 7 for two weeks). In all cases, the condition had worsened or shown no improvement in response to this treatment, leading to the decision to administer intracameral voriconazole injections. Four patients had developed imminent corneal perforations during topical amphotericin B treatment, which were treated with cyanoacrylate glue and bandage contact lenses (Cases 1, 2, 4, and 5). In Case 1, we removed the hypopyon due to its large volume. Four patients received one intracameral voriconazole injection (Cases 1, 2, 3, and 7), two patients received three injections (Cases 4 and 5), and one patient received four injections (Case 6). There were no perioperative complications associated with the injection procedure. After receiving intracameral voriconazole, the infection was eradicated in four patients, with a mean healing time of 7.5 weeks from the onset of symptoms (Cases 3-6). The remaining three patients required therapeutic keratoplasty (Cases 1, 2, and 7). In two of these, therapeutic keratoplasty was recommended due to the development of large corneal perforations (Cases 1 and 2). In the third case, keratoplasty was required due to treatment failure (Case 7). Of the three eyes that underwent keratoplasty, two were infected with *Fusarium* spp., with severe inflammation that quickly progressed to corneal melting (Cases 1 and 7). In these cases, major keratoplasty was required to preserve the anatomical integrity of the eye. No recurrence was observed in any of the patients during the follow-up period.

**Table 1:** Patient characteristics

	Age/Sex	Presenting BCVA	Presenting clinical features	Previous medications	History	Duration of symptoms	Culture	Confocal microscopy	N^o^ of injections	Additional treatment given	Final outcome	Final BCVA	Follow-up
Case 1	37/M	Light perception	Ulcer 6 × 7 mm, full-thickness infiltrate, endothelial plaque, hypopyon+	Moxifloxacin, cefalotin	Vegetative trauma	2 months	Fusarium solani	-	01	AMB; TA; BCL	TK	Counting fingers	3 years
Case 2	32/M	20/80	Ulcer 3 × 2.5 mm, full-thickness infiltrate	Moxifloxacin	-	2 weeks	Negative	Pseudohyphae	01	AMB; TA; BCL	TK	Counting fingers	3 years
Case 3	41/M	Hand motion	Ulcer 2 × 4 mm, peripheral vascularization, deep stromal infiltrate	Acyclovir oral, moxifloxacin, tobramycin e cefalotin	Foreign body	3 weeks	Scopulariopis brevicaulis	-	01	AMB	Vascularized cornea scar	Light perception	6 months
Case 4	74/F	Light perception	Ulcer 4 × 8 mm, full-thickness infiltrate, endothelial plaque	Gatifloxacin, gentamycin e cefalotin	Advanced glaucoma	1 month	Negative	Fungal hyphae	03 (72 h apart)	AMB; TA; BCL	Vascularized cornea scar	No light perception	4 months
Case 5	61/F	Light perception	Full-thickness infiltrate, hypopyon+	Moxifloxacin, prednisolone acetate	Prior keratoplasty	1 month	Candida parapsilosis	-	03 (72 h apart)	AMB; TA; BCL	Vascularized cornea scar	Counting fingers	2 years
Case 6	79/F	Hand motion	Ulcer 3 × 2 mm, hypopyon+, full-thickness infiltrate, endothelial plaque	Moxifloxacin, vancomycin, timolol, dorzolamide	Vegetative trauma	1 month	Negative	Fungal hyphae	04 (72 h apart)	AMB	Vascularized cornea scar	Hand motion	7 months
Case 7	48/M	Hand motion	Ulcer 6 × 6 mm, hypopyon+, full-thickness infiltrate endothelial plaque	Natamycin, moxifloxacin, timolol	Vegetative trauma	3 weeks	Fusarium solani	-	01	AMB	TK	Hand motion	1 year

AMB= topical 0.15% amphotericin B; BCL= bandage contact lens; BCVA= best-corrected visual acuity; F= female; M= male; TA= cyanoacrylate tissue adhesive; TK= therapeutic keratoplasty.

**Figure 1:**
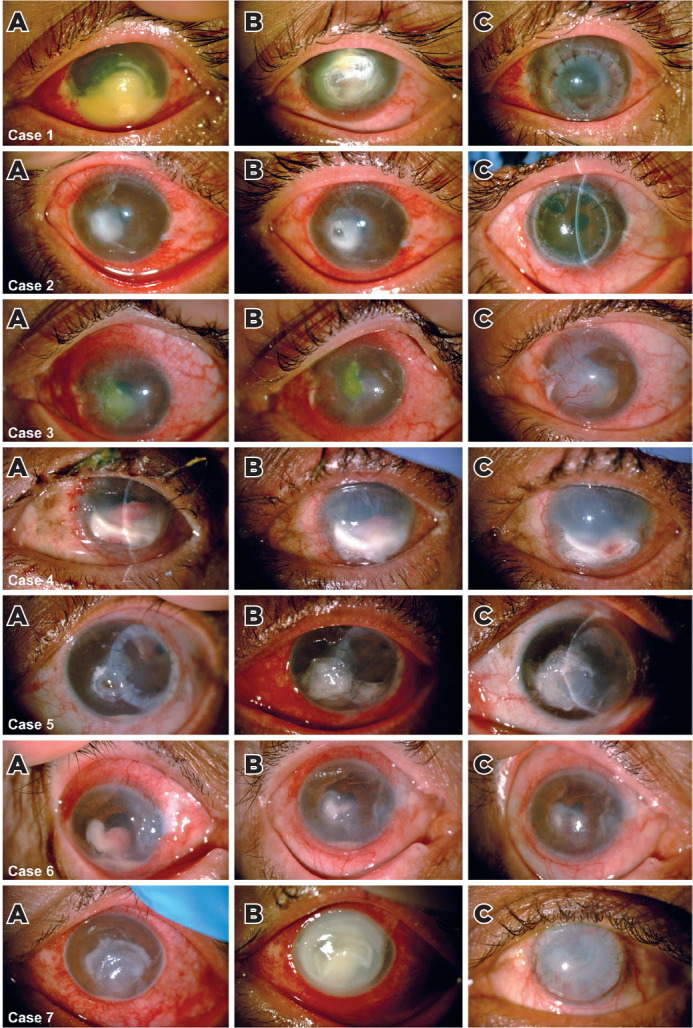
Slit-lamp photographs of patients with severe fungal keratitis (Cases 1 to 7) (A) before; (B) during; and (C) after treatment with intracameral voriconazole

At presentation, best-corrected visual acuity (BCVA) varied from 20/80 in only one patient to hand motion or worse in the other six. In two cases, the final BCVA had progressed from light perception to finger counting (Cases 1 and 5). In the remaining five eyes, there was no improvement in BCVA. All patients had a final BCVA of counting fingers or worse. Figure 1 shows the clinical progression of each patient.

## DISCUSSION

With the antifungal drugs currently available, there is no ideal treatment for deep fungal keratitis, particularly when the infection worsens despite topical treatment. Therefore, due to the lack of previous publications on this topic, this paper provides a description and discussion of our clinical experience with intracameral voriconazole as an adjuvant treatment for severe fungal keratitis. Treatment of ocular fungal infections is challenging due to pathogen diversity, delays in diagnostic confirmation, and the low effectiveness of available antifungals^(11)^. The most common diagnostic procedure is corneal scraping for smears and cultures. The sensitivity of the subsequent tests varies (smears between 27-94% and cultures between 43-97%)^(12^-^15)^. Polymerase chain reactions and confocal microscopy are useful diagnostic tools but are not widely available. The culture sensitivity rate in our series was 57%, which is consistent with the literature^(13^-^15)^. Confocal microscopy was used as a supplementary approach in cases with clinical signs of fungal infection but negative cultures.

Among our patients, trauma caused by organic material was the leading cause of fungal keratitis, with filamentous fungi the most common type identified in cultures and suspected by confocal microscopy. This is concordant with the epidemiological findings for our country (Brazil)^(2^,^16)^. Because filamentous fungi can infect deep stromal layers and penetrate an intact Descemet’s membrane, anterior chamber involvement, such as endothelial plaque and hypopyon, is not uncommon. Given the poor corneal penetration capacities of antifungal drugs, the challenge in these cases is to achieve sufficient intraocular concentrations. To this end, alternative routes of administration (subconjunctival, intrastromal, or intracameral) may be used^(8^,^15^,^17)^. Intracameral amphotericin B (AMB) has been used as adjuvant treatment in such cases with good results. However, it has been associated with adverse effects^(17^,^18)^.

Amphotericin B is a polyene macrolide that increases cell permeability by binding to ergosterol. It is the first broad-spectrum antifungal drug identified^(17)^. In ophthalmology, it is the first-line treatment for infections with yeast and natamycin-resistant filamentous fungi, particularly *Aspergillus*. It is less effective against *Fusarium* spp.^(16)^ *In vitro* studies have found varying levels of efficacy for amphotericin B, with minimum inhibitory concentrations (MICs) ranging between 0.5-6.73 µg/mL for *Aspergillus* spp. and *Fusarium* spp. and, for *Fusarium solani*, between 1.56-100 µg/mL^(18^-^20)^. Topical amphotericin B (in formulations of 0.15-0.5%) is well tolerated and is frequently used as the first-line treatment for deep fungal keratitis. It is preferred to natamycin as natamycin molecules are large with low water solubility and corneal penetration. Hence, natamycin is recommended as a monotherapy for superficial fungal infections^(17)^. Also, natamycin is currently formulated as a suspension, preventing its targeted delivery by injection^(8^,^17)^.

Topical amphotericin B penetration is also low in patients with an intact corneal epithelium; nonetheless, periodic debridement of the corneal epithelium helps to achieve therapeutic levels of penetration in the corneal stroma^(17)^. Subconjunctival administration is limited because of the potential risks of conjunctival necrosis, scleritis, and scleral thinning^(17)^. Nevertheless, intracameral, intrastromal, and intravitreal administration of amphotericin B have been used as alternative routes of administration in the treatment of deep keratomycosis and endophthalmitis. This has resulted in favorable outcomes and faster healing^(7^,^8^,^21^-^23)^. However, pain, toxicity, inflammation, corneal edema, anterior chamber reactions, iritis, cataracts, and retinal necrosis have all been reported as complications of this approach^(17)^.

Voriconazole is a third-generation azole that inhibits fungal cytochrome P450 enzymes by blocking ergosterol synthesis in the plasma membrane. It has a higher efficacy against filamentous fungi and lower MICs than first-generation azoles. *In vitro* studies indicate that voriconazole has a broader spectrum and higher efficacy against *Candida* spp. and *Aspergillus* spp. It has a similar MIC to amphotericin B against *Fusarium* spp^(17^,^24)^. The voriconazole MIC for *Candida* spp. ranges between 0.06-0.25 µg/mL; for *Aspergillus* spp., the MIC is 0.5 µg/mL; and for *Fusarium oxysporum* and *Fusarium solani*, the MICs ranges between 2-8 µg/mL^(24^-^27)^. Shen et al.^(28)^ found that voriconazole is eliminated more quickly from the anterior chamber than from the vitreous, with a half-life of 22 min in the anterior chamber of rabbits. However, intracameral voriconazole is the most effective method of increasing aqueous concentrations^(18)^. Despite its rapid elimination, 50 µg/0.1 mL is much higher than the MICs for *Candida, Aspergillus*, and *Fusarium* spp. Therefore, repeated voriconazole injections appear to be necessary. Also, previous studies have found it an effective alternative treatment option for deep keratomycosis^(3^,^8^,^18)^.

Despite targeted voriconazole treatment, all of our patients had unsatisfactory BCVA outcomes. The literature has shown that diagnostic delay, infection severity at presentation, and deep infiltrates are all associated with a worse visual prognosis^(29)^. It should be noted that all patients but one had visual impairment at presentation due to underlying conditions and infection severity. Fungal eradication was successful in all cases and there was no recurrence during the follow-up, either in those responsive to the intracameral voriconazole or those who required keratoplasty. Nevertheless, the final visual acuity of all seven patients was poor, highlighting the serious nature of fungal keratitis.

We saw no perioperative complications or adverse effects after the intracameral voriconazole injections. This is consistent with previous *in vitro* and *in vivo* safety and low toxicity findings^(30)^. The preparation of the voriconazole used with our sample by a pharmaceutical company ensured that it was safe for administration. It also aimed to standardize the treatment and reduce costs, as the price of voriconazole typically limits its use, particularly in low-income countries with public healthcare, such as Brazil. Three of the seven cases underwent multiple injections, resulting in fungal eradication. Four patients received only one injection. Of these, the infection was resolved in one, while the other three required therapeutic keratoplasty. Keratoplasty was recommended after worsening of the keratitis, with corneal perforation or persistent infection. Two of the three eyes that underwent keratoplasty were infected with *Fusarium* spp., causing severe corneal melting. *Fusarium* spp. are the leading cause of corneal transplantation requirements in patients with fungal keratitis^(29)^. These fungi are particularly challenging to treat due to resistance and the need for high antifungal concentrations. *Fusarium* keratitis often progresses to perforation, endophthalmitis, and enucleation without adequate treatment. It is also associated with recurrence after keratoplasty^(29)^. As this was a case series, our results do not guarantee a reduced need for therapeutic grafts or the prevention of more severe outcomes with intracameral treatment. However, among our sample, intracameral voriconazole injections were a safe and beneficial treatment for deep fungal keratitis that was worsening with conventional treatment. Nevertheless, it is important to emphasize the need for comparative studies with larger samples to confirm our findings.

In conclusion, intracameral voriconazole injections are a feasible adjuvant treatment for deep fungal keratitis. However, there is limited clinical evidence of their efficacy and safety, which mainly comes from case reports and case series. Therefore, randomized clinical trials with larger sample sizes are warranted.
